# Operative Surgery and Management

**Published:** 1981

**Authors:** 


### Book Review

**OPERATIVE SURGERY AND MANAGEMENT**
**Edited by G. Keen**
**John Wright, Bristol**
**£25**

This book is a considerable triumph for its editor and principal author Gerald Keen. He has had at the outset two big judgements to make and he has got them both about right – how large to make it? – it is about as large a single volume as you can comfortably hold, and how far afield to go for contributors? Of 54 authors, 24 are from Bristol and 12 of these are from Frenchay Hospital. But he has also gone to acknowledged masters elsewhere in this country and also to the USA which provides 8 of his authors. In so doing he has produced a work with a strong Bristol accent and yet with an international authority.

The complete field of operative surgery is covered with the exception of ENT and Ophthalmic surgery.

The book is intended for trainees and residents in general surgery but it will certainly be widely used by established surgeons approaching unfamiliar territory. Especially is such a book invaluable to the surgeon in isolated areas who has to tackle whatever problems come in through the door.

A special word of praise must be said about the format and the quality of the illustrations, both of which are most elegant. It is also at present day prices for such a large and beautiful book a bargain at £25.

This book can only enhance the reputation of Bristol as a centre of surgical excellence, and for the contributors, to paraphrase Dr. Johnson, it will have concentrated their minds wonderfully.

